# Facile Synthesis of Cyclic Polyamidine with High Cationic Degree Using Environmentally Benign Approach

**DOI:** 10.3390/molecules28062530

**Published:** 2023-03-10

**Authors:** Bo Guo, Xinghua Teng, Xiaohan Guo, Sen Zhang, Leilei Wang, Jianing Wang

**Affiliations:** 1 Shandong Institute of Metrology, Jinan 250014, China; 2 Shandong Province Key Laboratory of Applied Microbiology, Ecology Institute of Shandong Academy of Sciences, Qilu University of Technology (Shandong Academy of Sciences), Jinan 250014, China

**Keywords:** cyclic polyamidine, polymerization and hydrolytic amidinization reaction, environmentally benign approach, supercritical carbon dioxide

## Abstract

An environmentally benign approach was developed to fabricate cyclic polyamidine via polymerization in supercritical carbon dioxide (SCCO_2_) and subsequently amidinization in water. Synthetic parameters were evaluated using response surface methodology. In comparison with aqueous solution polymerization for the fabrication of PNVF-co-PAN, polymerization using SCCO_2_ is favorable to promote the yield and viscosity of PNVF-co-PAN and diminished reaction time on account of excellent solvation capacity and transport property of SCCO_2_. Replacing the traditional water solution with SCCO_2_ as a green solvent could heighten the purity of PNVF-co-PAN by virtue of the excellent extraction of SCCO_2_. The cationic degree (5.66 mmol/g) of polyamidine fabricated by environmentally benign approach was significantly higher than that in previous reported studies.

## 1. Introduction

Polyamidine, one class of macromolecular polymer comprising an amidine unit in its backbone structure, can be divided into two classes: polyamidine containing linear-chain amidine groups and polyamidine containing cyclic amidine groups [1,2,3]. In comparison with liner polyamidine, cyclic polyamidine has received significant attention owing to its high molecular weight, outstanding positive-charge density, and good thermal stabilities [4]. These unique properties make cyclic polyamidine ideal candidates as cationic flocculants in wastewater treatment [5,6]. The emergence of facile, efficient and environmentally benign approach for preparation of cyclic polyamidine is highly desirable, owing to its broad application and enormous demand.

The synthetic approach for cyclic polyamidine is divided into two parts: the polymerization reaction and the hydrolytic amidinization reaction. In the polymerization stage, N-vinylformamide (NVF) and acrylonitrile (AN) are used as monomers, and the corresponding copolymerization product (PNVF-co-PAN) is obtained through aqueous solution copolymerization reaction in the presence of initiators, which are frequently classified into two types: inorganic initiators and organic initiators [7]. In the hydrolytic amidinization reaction, the amide group of PNVF-co-PAN is hydrolyzed under acidic conditions, giving rise to the amine unit. This amine group further reacts with adjacent cyano groups to produce polyamidine with a five-membered ring structure [4]. Over the past decade, many efforts have been concentrated on the preparation of cyclic polyamidine. Shinichi Sato et al. fabricated cyclic polyamidine via the aqueous solution polymerization of NVF with AN and amidinization under acidification [5]. The results showed that the sludge dehydration efficiency of the cyclic polyamidine was outstanding. Wang et al. prepared the polyamidine firstly through the aqueous solution polymerization of NVF with AN within China [8]. The properties of polyamidine were assessed in terms of charge density, viscosity, and flocculating effect. The structure of the polyamidine was characterized by FT-IR and UV. Zhao et al. proposed a cationic starch-grafted polyamidine as a flocculant, and investigated its application in oil–water treatment [9]. The starch-grafted intermediate was fabricated by adding starch as a matrix and crosslinker in the aqueous solution polymerization of NVF with AN, and subsequently amidinized under acidic conditions to generate starch-grafted polyamidine, which possessed a higher molecular weight and inexpensive cost. The experimental results indicated that the starch-grafted polyamidine exhibited promising oil–water separation efficiency. However, the synthetic pathway for preparing cyclic polyamidine based on aqueous solution polymerization may lead to lower yields and unsatisfactory charge density of the corresponding product [8,10]. Water used in polymerization should be further treated to prevent environmental pollution. Therefore, developing facile, efficient, and environmental benign approaches for the fabrication of cyclic polyamidine still remains of considerable significance.

In recent years, supercritical carbon dioxide (SCCO_2_) has been applied as an environmentally benign solvent substitute to develop the synthetic strategy of materials and pharmaceuticals, in view of SCCO_2_’s advantages, such as mild critical conditions, stable properties, fast mass transfer rates, and easy isolation and purification of reaction products [11,12,13]. Much effort has been invested in polymerization using SCCO_2_ as a dispersion solvent [14,15,16]. The copolymer with a suitable polymerization degree, narrow molecular distribution and high yield was obtained through synthesis technology in SCCO_2_. The unreacted monomers, oligomers, and initiators were dissolved readily in SCCO_2_ owing to the excellent extraction of SCCO_2_. The above SCCO_2_ solution was drawn from the polymerization system, facilitating the purification of copolymers. Saraf et al. prepared poly(1,1-difluoroethene) via precipitation polymerization using SCCO_2_ as a solvent and investigated its molecular weight distribution [17]. The reaction parameters for the precipitation polymerization of 1,1-difluoroethene in SCCO_2_ and the polymerization model were investigated in-depth by Mueller et al. [18]. Cooper et al. synthesized highly cross-linked poly(divinylbenzene) microspheres in SCCO_2_ by precipitation polymerization [19]. Yoshida et al. prepared copolymer microspheres by coprecipitation polymerization of fluorinated acrylate with 2-(dimethylamino) ethyl acrylate in SCCO_2_ [20]. However, PNVF-co-PAN, an important intermediate polymer for the fabrication of polyamidine, was rarely synthesized via polymerization in SCCO_2_. In addition, the effect of SCCO_2_ on the removal of reaction monomer and initiator, as well as the improvement of PNVF-co-PAN purity, needs further investigation.

**Scheme 1:**
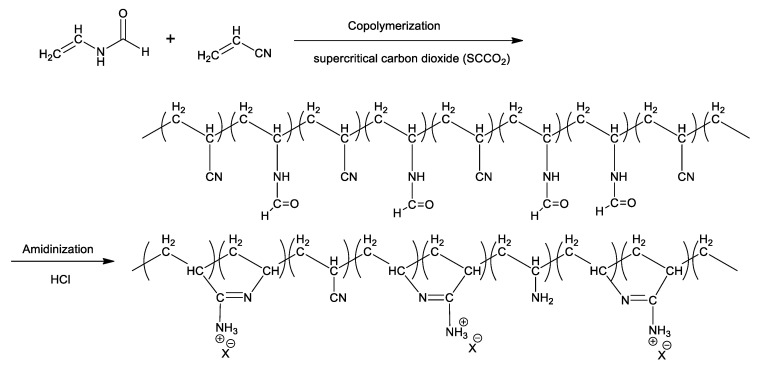
Synthesis process of cyclic polyamidine.

In this study, an environmentally benign approach was developed to fabricate cyclic polyamidine via polymerization in SCCO_2_ and subsequently amidinization in water (Scheme 1). The parameters influencing polymerization and hydrolytic amidinization were separately evaluated. We focused on the effects of three parameters, including reaction temperature, reaction pressure, and initiation dose, on the synthesis of PNVF-co-PAN via polymerization reaction in SCCO_2_. The influences of amidinization temperature, hydrochloric acid dose, and amidinization time on the yield and charge density of polyamidine were analyzed. Response surface design experiments were utilized to identify the optimal conditions of polymerization and amidinization, respectively.

## 2. Results and Discussion

### 2.1. Optimization of Polymerization Conditions for PNVF-co-PAN

**Figure 1:**
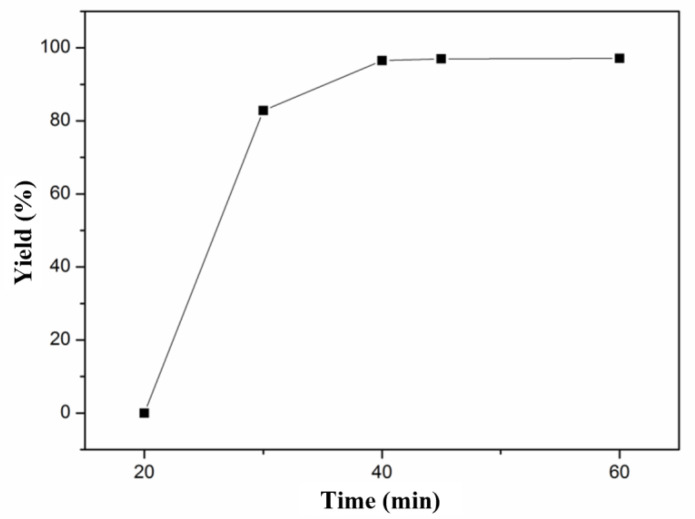
Effect of SCCO_2_ copolymerization time on yield.

According to our published results [4], PNVF-co-PAN was constructed through the aqueous solution polymerization of NVF and AN with a molar ratio of 1:1 using dihydrochloride (AIBA) initiation at 50 °C for 5 h. The molar ratio (1:1) and a mass fraction (0.15) of monomers were employed in the SCCO_2_ synthetic strategy of polyamidine, and other copolymerization parameters, such as reaction temperature (60 °C), reaction pressure (15 MPa), and initiator dosage (AIVN, 0.015 g), were chosen to explore the effect of reaction time on the yield. As illustrated in Figure 1, no product appeared in at the initial 20 min, but a yield of 85% was obtained at 30 min. The yield increased slightly from 95% to 97% with the increase in reaction time from 40 min to 60 min. The results reveal that the reaction was basically completed in this system after 40 min. In comparison with copolymerization in water, the reaction time of copolymerization in SCCO_2_ was decreased considerably due to the excellent mass transfer capability of SCCO_2_. To ensure that copolymerization in SCCO_2_ was complete, 1 h was chosen as the specified reaction time for the following experiment.

**Figure 2:**
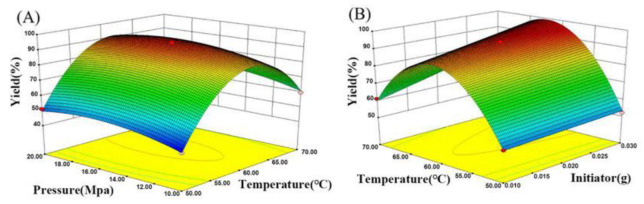
(**A**) Response surface on yield versus temperature and pressure; (**B**) response surface on yield versus temperature and initiator.

**Figure 3:**
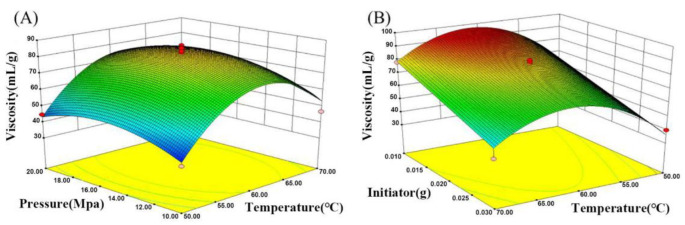
(**A**) Response surface on viscosity versus temperature and pressure; (**B**) response surface on viscosity versus temperature and initiator.

The 3D response surface methodology (RSM) model, as a collection form for experimental data using a multivariate mathematical-statistical tool, was plotted to visualize the influence between the response (i.e., the yield and viscosity of polyamidine) and any two independent parameters (i.e., temperature as X1, pressure as X2, and initiator dose as X3) in supercritical polymerization. Box–Behnken design was applied in this optimization process, and the corresponding non-coded level of parameters and 17 experimental runs were given in Tables S1 and S2. Using the Box–Behnken design, the models’ fitness was evaluated, and response surface plots (Figure 2 and Figure 3) were obtained by utilizing the statistical software package Design-Expert 8.0.5. Analysis of variance (ANOVA) was used as the indicator to assess the quality of these fitted models, and the results were tabulated in Tables S3 and S4. Variance analysis was performed according to 5% confidence intervals. The well-known probability value (*p*-value) is less than 0.05, indicating the significance of the model. The data show that this test is credible and the model is reliable.

The effect of temperature on the yield during supercritical polymerization is shown in Figure 2. The yield increased gradually with rising temperature, and the maximum yield of 99% was achieved at 60.95 °C. However, the yield decreased when the temperature exceeded 60.95 °C. The result indicates that the decomposition of the initiator accelerated and the concentration of free radicals increased in the temperature rise period, resulting in faster reaction speed and higher yield. However, continuously increasing temperature may cause two unfavorable situations. Firstly, the reduction in mass transfer rate occurred in virtue of the continuously increasing temperature, resulting in a decrease in the polymerization speed. Secondly, a large amount of initiator was consumed due to the rapid polymerization of monomers and precipitation of corresponding copolymer at higher temperatures. Polymerization no longer proceeded once the concentration of the initiator dropped below a certain amount, leading to a decrease in yield.

The effect of pressure on the yield during supercritical polymerization is illustrated in Figure 2A. The yield rose continuously when the pressure increased from 10 MPa to 15.29 MPa because the SCCO_2_ density increased at relatively higher pressure, giving rise to the enhancement of mass transfer capacity and the acceleration of the reaction rate. The maximum yield of 99% was present at 15.29 MPa. However, the yield decreased slightly with a further increase in pressure, because enhancing the pressure can raise the temperature, causing a decrease in SCCO_2_ density.

The effect of initiator dosage on yield during supercritical polymerization is also displayed in Figure 2B. The yield increased with the increment of initiator dosage from 0.015 g to 0.025 g. Elevating the initiator dosage caused the amplification of free radicals and an acceleration of the reaction rate, resulting in a higher yield. A fast mass transfer and no hindrance of motion existed in SCCO_2_. Conversely, owing to the fast polymerization, the solution viscosity was intensified in aqueous solution polymerization, hindering the movement of the monomer and the initiator, which may retard the yield.

ANOVA demonstrated that the *p*-values of temperature, pressure, and initiator dose were all less than 0.0001, which implies that the combined interaction among extraction temperature, pressure, and initiator dosage influenced the final yield of polyamidine.

Figure 3 illustrates the response surface graph about the effect of three factors (e.g., temperature, pressure) on viscosity during supercritical polymerization. The viscosity intensified gradually with a rise in temperature, reaching a maximum value of 97.34 mL/g at 61.38 °C, and viscosity decreased when the temperature exceeded 61.38 °C. The moderate rate of polymerization was obtained due to the proper decomposition of free radicals in the initial warming stage, resulting in the slow enhancement of solution viscosity. However, the excessive temperature may make the decomposition of the initiator accelerate and increase the number of active centers. The more active centers occur, the higher the content of shorter chain polymers, which leads to lower viscosity.

As shown in Figure 3A, pressure exhibited a small effect on viscosity. Nevertheless, the mass transfer rate was accelerated with an increase in pressure, which is conducive to the construction of long-chain polymers. Viscosity reached a maximum value of 97.34 mL/g when the pressure increased from 10 MPa to 15.52 MPa, and then decreased slightly as pressure further increased. The result was consistent with the above-mentioned results about the low yield under high pressure because rising pressure heightens the temperature of the system, causing the lower viscosity.

The effect of initiator dosage on viscosity during supercritical polymerization is represented on the response surface graph (Figure 3B). The viscosity decreased when the initiator increased from 0.015 g to 0.025 g. The use of plentiful initiators increased the concentration of free radicals and the number of shorter chain polymers. The lower degree of polymerization was attributed to the enormous shorter chain polymers prepared in the polymerization process, resulting in lower viscosity.

**Figure 4:**
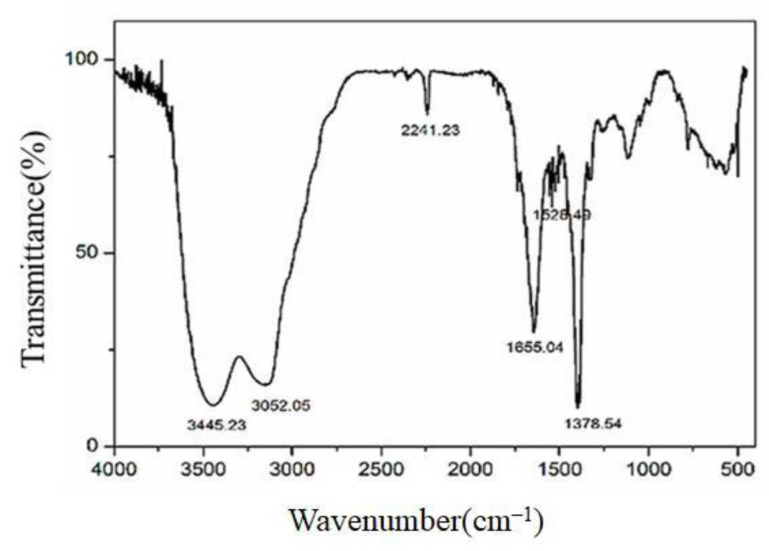
FT-IR spectrum of PNVF-co-PAN.

From the ANOVA data, it is clear that the effect of initiator dosage on viscosity is significant by virtue of the *p*-value of initiator dosage (0.0002). The increase in initiator dosage will decrease viscosity from 93.8 mL/g to 65 mL/g. The *p*-value of temperature was 0.0219, revealing that temperature was also a significant parameter. The viscosity increased from 52 mL/g to 85 mL/g with a rise in temperature. The *p*-value of pressure was 0.4749, which was greater than 0.05, indicating that pressure was an insignificant parameter. Considering the trend of yield and viscosity, the experimental parameters (temperature: 61.27 °C; pressure: 15.52 MPa, initiator dosage: 0.02 g) were adopted, and the best yield of 89% and maximum viscosity of 93.4 mL/g were obtained as the optimal conditions. FT-IR was used to characterize the functional groups on PNVF-co-PAN. As displayed in Figure 4, the stretching vibrations of cyano groups were located at 2241 cm^−1^, and the stretching vibrations of C=O bonds occurred at 1655 cm^−1^. The peak at 1378 cm^−1^ was assigned to the -C-N- stretching vibrations. The above results demonstrate that cyano groups and formamide groups existed in the PNVF-co-PAN backbone.

### 2.2. Optimization of Amidinization Conditions for Polyamidine

**Figure 5:**
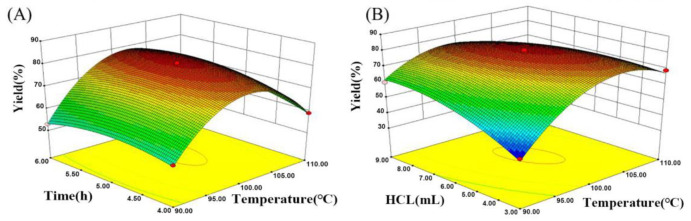
(**A**) Response surface for yield versus temperature and time; (**B**) response surface for yield versus temperature and dose of hydrochloric acid.

**Figure 6:**
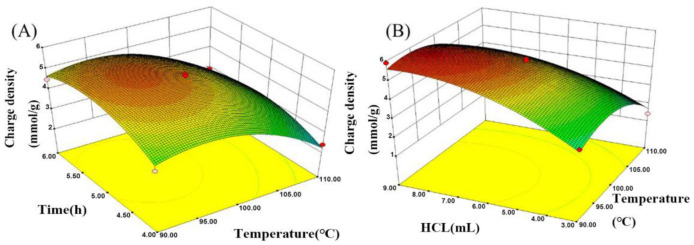
(**A**) Response surface on charge density versus temperature and time; (**B**) response surface on charge density versus temperature and dose of hydrochloric acid.

Three factors (e.g., temperature, time and HCl volume) were optimized using response surface methodology to obtain the optimal reaction conditions in the amidinization step. Each factor was tested at three levels (Table S5). Since yield is an important parameter reflecting the effectiveness of the amidinization approach, and a higher cationic degree of polyamidine could be conducive to enhance the applications performance as flocculant, the yield and cationic degree of polyamidine were adopted as dependent variables and expressed as Y1 and Y2, respectively. The Box–Behnken design was applied in this optimization process of amidinization approach, and 17 experimental runs were conducted as shown in Table S6. Figure 5 and Figure 6 illustrate the 3D response surface graphs based on the yield and cationic degree of polyamidine, respectively. ANOVA (Table S7) was used to assess the suitability and significance of the 3D model. The *p*-values of the three factors were all < 0.0500, which implies that these model terms were notable. These findings suggest that the established 3D models for seeking optimum amidinization conditions were dependable.

Figure 5A illustrates the effect of temperature and time on the yield during amidinization process. The yield of polyamidine increased as the temperature increased from 90 °C to 101.1 °C, reaching a maximum of 80.9%. The yield decreased sharply to 66.0% with a rise in temperature from 101.1 °C to 110 °C. This phenomenon can be explained as a result of the following: to facilitate the formation of cyclic polyamidine by using amidinization between the amine groups and adjacent cyano group, amide units introduced in the copolymer were hydrolyzed to amine groups under acidic conditions. Within a certain range, an increase in temperature accelerated the hydrolysis process, facilitating the formation of amine groups. When the temperature exceeded 100 °C, a water-immiscible side product was prepared by a cross-linking reaction between amine groups on the different polymer chains, which restrained the formation of amine groups present on the main chain, causing a lower yield. Hence, 100 °C was adopted as a suitable amidinization temperature.

Figure 5A displays that the reaction time exhibited a positive impact on the yield. The productivity of polyamidine became evident after 4 h and subsequently reached a maximum value at 5 h. The stable yield was discovered at the time of 5–6 h. Therefore, 5 h was considered the best reaction time.

Figure 5B indicates the effect of temperature and the HCl volume on the yield during the amidinization process. The results show that the yield increased and reached a maximum of 81.1% when the HCl volume was increased from 3 mL to 5.96 mL. Then, the yield showed a faint change as the HCl volume was raised from 5.96 mL to 9 mL and was reduced to 76% when the volume of hydrochloric acid was 9 mL. The trend of yield increased initially and became unvarying with the rise in HCl volume. The reason for this phenomenon is that the hydrolysis reaction can be carried out under acidic conditions, and the increase in hydrogen proton improves the reaction rate of hydrolysis. When the HCl volume continued to increase, the stable yield was obtained in virtue of the completeness of hydrolysis reaction. The side product was synthesized through the hydrolysis of cyano groups under strongly acidic conditions. The substantial consumption of cyano groups retarded the formation of polyamidine. So, 7 mL was selected as the optimum volume in view of the response surface model.

The effects of temperature and time on the cationic degree are presented in Figure 6A. The results imply that the cationic degree of polyamidine was heightened from 5.08 mmol/L to 5.85 mmol/L when the temperature was raised from 90 °C to 95 °C. Then, increasing the temperature from 95 °C to 110 °C retarded the cationic degree to 3.15 mmol/L. The trend of temperature on the cationic degree was analogous to the trend of temperature on yield in amidinization, primarily because the yield was declined on account of the cross-linking reaction between amine groups at higher temperatures, resulting in a lower cationic degree. Hence, a relatively moderate temperature was chosen to generate a higher cationic degree.

Figure 6A indicates that the effect of reaction time on the cationic degree was in line with the effect of reaction time on the yield in the amidinization process. The cationic degree was elevated and reached a maximum when the reaction time was increased from 4 h to 5.2 h. Then, the cationic degree showed a slight variation with the rise in reaction time (from 5 h to 6 h). Therefore, 5 h was selected as the most favorable reaction time.

The effect of temperature and HCl volume on the cationic degree during amidinization process is illustrated in Figure 6B. The results imply that the cationic degree was raised with increase in HCl volume and reached a maximum of 5.85 mmol/L when the volume of HCl was 5.96 mL. When the HCl volume was increased up to 9 mL, the cationic degree was slightly reduced. The trend of HCl volume on the cationic degree was consistent with the trend of HCl volume on yield in the amidinization process. The content of amine group formed via the hydrolysis reaction was heightened with the rise in the concentration of hydrogen proton, resulting in the superior cationic degree. The invariable cationic degree was achieved due to the termination of the hydrolysis reaction. The hydrolysis of cyano groups suppressed the formation of five-membered rings comprising amidine group when acidity reached a certain higher level, leading to a reduction in the cationic degree.

According to the response surface model, the optimal conditions were considered as follows: the amidinization temperature was 98 °C, the time was 5.0 h, and the volume of HCl was 7.4 mL. Characterization data of polyamidine prepared at the above optimal conditions were listed as follows: the yield was 79.4%, Mn was 6.31 × 10^5^ Da, Mw was 6.59 × 10^5^ Da, the polydispersity index (PDI) was 1.04, the cationic degree was 5.66 mmol/g, and the viscosity was 304.2 mL/g.

## 3. Materials and Methods

### 3.1. Reagents

N-vinylformamide (NVF) was obtained from TCI (Tokyo Chemical Industry Co., Ltd., Shanghai, China). Acrylonitrile (AN) was purchased from Sinopec Qilu Petrochemical Company (Zibo, China). 2,2′-Azobisisoheptonitrile (AIVN) was purchased from Xiya Reagent Co., Ltd. (Linyi, China). Hydrochloric acid (HCl) and acetone were obtained from Sinopharm Chemical Reagent Co., Ltd. (Shanghai, China). Carbon dioxide (CO_2_), with a 99.9% minimum purity, was purchased from Jinan Deyang Special Gas Co., Ltd. (Jinan, China).

### 3.2. Copolymerization

A known amount of a mixture comprising NVF and AN in a molar ratio of 1:1 was injected into the high-pressure vessel. N_2_ was bubbled into the high-pressure vessel for 15 min. AIVN (at 0.3 wt.% with respect to the amounts of loaded monomers, dissolved in 5 mL methanol) was added dropwise slowly to the high-pressure vessel under an N_2_ atmosphere. The high-pressure vessel was sealed and purged with carbon dioxide to remove oxygen. The reaction mixture was pressurized with the required pressure (10–25 MPa) and heated to the desired temperature (50–70 °C) for a reaction time varying from 20 min to 60 min. After the copolymerization was complete, the CO_2_ was immediately vented. The resulting PNVF-co-PAN was obtained.

### 3.3. Amidinization

The copolymers after copolymerization were dissolved in HCl (0.5–1.5 equivalent of the total moles of loaded monomers) and ultrapure water (3 times the total volume of the loaded monomers), and then the reaction mixture was heated up to 90–110 °C for 4–6 h. After the amidinization was complete, the reaction mixture was cooled and poured into acetone. The resulting polyamidine was precipitated and dried at room temperature under vacuum until constant weight was reached.

### 3.4. Evaluation of Yield

The synthetic process consists of a polymerization reaction and an amidinization reaction. The yield was expressed as the yield of the polymerization reaction (the first step) and the yield of the amidinization reaction (the second step).

The polymerization reaction yield (*Yield*_1_) was calculated according to the following equation:

(1) $${Yield}_{1}=\frac{m_{1}}{M_{1}}\times 100\%$$

where *m*_1_ is the mass of polymer, and *M*_1_ is the total mass of two monomers.

The amidinization reaction yield (*Yield*_2_) was calculated according to the following equation:

(2) $${Yield}_{2}=\frac{m_{2}}{M_{T}}\times 100\%$$

where *m*_2_ is the mass of polyamidine, and *M_T_* is the theoretical mass of the polyamidine calculated based on the molar fraction of the two monomers.

### 3.5. Viscosity Measurement

The relative viscosity was measured using a Ubbelohde viscometer placed in a water bath at 20 °C. The copolymer (0.1 g) was dissolved in DMSO (100 mL). A certain amount of the above copolymer solution was added to the Ubbelohde viscometer, then the system was incubated in a water bath for 20 min. The intrinsic viscosity of the copolymer sample was determined by extrapolation. The measurement of polyamidine’s viscosity was consistent with the above process, except that replace DMSO with water, which was used to dissolve the polyamidine.

### 3.6. Cationicity Measurement

The cationicity of polyamidine was measured by the titration method with an indicator. A 10 mL solution of polyamidine (1 g/L) was diluted with water to 100 mL in a conical flask. Then, indicator (T.B.) was added to the above polyamidine solution. The standard solution of potassium poly(vinylsulfate) (PVSK) was used to titrate the polyamidine solution. The amount of PVSK solution consumed was recorded when the solution turned from blue to purple. The blank group was measured as above route. Cationicity was calculated according to the following equation:

(3) $$C\mathrm{ationicity}=\frac{C\;\times \;(V-V_{0})}{M_{2}}$$

where C is the concentration of PVSK solution (mol/L), V is the volume of PVSK solution (mL), *V*_0_ is the volume of PVSK solution consumed in blank titration (mL), and *M*_2_ is the mass of polyamidine.

### 3.7. FT-IR Spectroscopy

With powdered sample being pressed into a piece, the FT-IR spectra of the copolymer was recorded with a PerkinElmer “Spectrum BX” FT-IR spectrometer in the range of 4000–400 cm^−1^.

### 3.8. Molecular Weights Measurement

Molecular weights (Mn and Mw) and molecular weight distributions (Mw/Mn) were determined by size exclusion chromatography (SEC). SEC analyses were carried out using a GPC/SEC system (PL-GPC 50, Agilent) with a refractive index detector (RI) and a UV detector. The separation was performed using a Shodex OH-PAK SB-806 column. The eluent was a mixture of ultrapure water containing 1.17 wt% sodium chloride and 0.02 wt% sodium azide. The flow rate was 0.5 mL/min.

## 4. Conclusions

Cyclic polyamidine was synthesized using NVF and AN as raw materials through the environmentally benign approach, which consisted of polymerization in SCCO_2_ and hydrolytic amidinization in water. The results imply that the synthetic parameters, such as temperature, pressure, and initiator dosage, were crucially beneficial for affecting yield and viscosity in supercritical polymerization. In comparison with aqueous solution polymerization, the copolymer obtained in SCCO_2_ exhibited high yield and satisfactory viscosity on account of the excellent solvation capacity and transport property of SCCO_2_. Replacing the traditional water solution with SCCO_2_ as a green solvent could heighten the purity of PNVF-co-PAN by virtue of the excellent extraction of SCCO_2_. The influence of amidinization parameters, such as temperature, time, and HCl volume, on the yield and cationic degree of polyamidine was examined. After optimization, the optimum conditions for the fabrication of polyamidine were achieved. The cationic degree (5.66 mmol/g) of polyamidine prepared at the optimum condition was significantly higher than that in previous reported studies. The higher yield, satisfactory viscosity, and outstanding cationic degree of polyamidine prepared through an environmentally benign approach made it possible to exhibit exceptional performance as a flocculant in the environmental application of sludge dewatering.

## Data Availability

Not applicable.

## Supplementary Materials

The following supporting information can be downloaded at: https://www.mdpi.com/article/10.3390/molecules28062530/s1, Table S1. Range of independent variables in the SCCO_2_ copolymerization for the B-B method; Table S2. Range of independent variables in the SCCO_2_ copolymerization for the B-B method; Table S3. *p*-value of yield in SCCO_2_ copolymerization; Table S4. *p*-value of viscosity in SCCO_2_ copolymerization; Table S5. Range of three independent variables in amidination for the B-B method; Table S6. Range of three independent variables in amidination for the B-B method; Table S7. *p*-value of yield and charge density in in amidination.
