# Supplementary material for: Facile Synthesis of Cyclic Polyamidine with High Cationic Degree Using Environmentally Benign Approach
Source: Molecules. 2023 Mar 10;28(6):2530. doi: 10.3390/molecules28062530 (PMC10054134; doi:10.3390/molecules28062530)
Supplement: Supplementary file 1 [file molecules-28-02530-s001.zip › molecules-2184038-supplementary.pdf]

Supplementary Materials

Facile Synthesis of Cyclic Polyamidine with High Cationic Degree using Environmentally Benign Approach

Table S1. Range of independent variables in the $SCCO_2$ copolymerization for the B-B method

Independent variable	Level		
	-1	0	1
A Temperature (°C)	50	60	70
B Pressure（MPa）	10	15	20
C Initiator dose（g）	0.015	0.020	0.025

Table S2. Range of independent variables in the $SCCO_2$ copolymerization for the B-B method

Run number	Temperature ℃	Pressure MPa	Initiator dose g	Yield %	Viscosity mL/g
1	60	20	0.025	95.1	56.7
2	60	15	0.02	92.1	85
3	60	15	0.02	93	82.7
4	60	15	0.02	92.7	85.3
5	50	20	0.02	51.3	44.8
6	70	15	0.025	77.8	42.6
7	50	15	0.025	52	39.8
8	60	15	0.02	93.8	83.4
9	50	15	0.015	51.4	62.3
10	60	10	0.025	91.4	57.4
11	70	20	0.02	67.3	71.6
12	60	15	0.02	94.2	82.7
13	60	20	0.015	84.1	77.4
14	60	10	0.015	79.3	93.8
15	50	10	0.02	43.9	37.4
16	70	10	0.02	59.9	46.7
17	70	15	0.015	61.2	78.2

Table S3. p-value of yield in $SCCO_2$ copolymerization

Factor	p-value（yield）	Significance
Model	< 0.0001	Significant
A- Temperature℃	< 0.0001	
B- Pressure MPa	< 0.0001	
C- Initiator dose g	< 0.0001	
AC	< 0.0001	
A^2	< 0.0001	
B^2	< 0.0001	
Lack of Fit	0.1741	Non-significant

Table S4. P-value of viscosity in $SCCO_2$ copolymerization

Factor	p-value （Viscosity）	Significance
Model	< 0.0001	Significant
A- Temperature℃	0.0219	
B- Pressure MPa	0.4749	
C- Initiator dose g	0.0002	
A^2	< 0.0001	
B^2	0.0242	
Lack of Fit	0.0009	

Table S5. Range of three independent variables in amidination for the B-B method

Independent variable	Level		
	-1	0	1
A Temperature (°C)	90	100	110
B Dose of hydrochloric acid（g）	3	6	9
C Time（h）	4	5	6

Table S6. Range of three independent variables in amidination for the B-B method

Run number	Temperature℃	Time h	Dose of hydrochloric acid mL	Yield %	Charge density ml/g
1	90	5	3	41	3.2
2	90	5	9	60.1	5.9
3	100	4	9	73.9	4.3
4	100	5	6	80.9	5.2
5	90	6	6	52.9	4.5
6	90	4	6	54.9	3.8
7	100	6	3	71.1	2.9
8	100	4	3	62.9	2.7
9	110	4	6	59.1	2.4
10	110	6	6	65.1	2.9
11	100	5	6	80.5	5.3
12	110	5	9	49.7	3
13	100	6	9	72.7	4.9
14	100	5	6	80.2	5.2
15	100	5	6	81	5
16	100	5	6	80.7	5.1
17	110	5	3	69	1.4

Table S7. *p*-value of yield and charge density in in amidination

Factor	*p*-value （Yield）	*p*-value（Charge density）	Significance
Model	< 0.0001	< 0.0001	Significant
A- Temperature℃	0.0003	< 0.0001	
B- Time h	0.0653	0.0324	
C- Dose of hydrochloric acid mL	0.0433	< 0.0001	
AB	0.0595	0.7179	
AC	< 0.0001	0.0773	
BC	0.0335	0.4763	
A^2	< 0.0001	< 0.0001	
B^2	0.0036	0.0009	
C^2	0.0001	0.0007	
Lack of fit	0.0002	0.02	
